# Respiratory Disease following Viral Lung Infection Alters the Murine Gut Microbiota

**DOI:** 10.3389/fimmu.2018.00182

**Published:** 2018-02-12

**Authors:** Helen T. Groves, Leah Cuthbertson, Phillip James, Miriam F. Moffatt, Michael J. Cox, John S. Tregoning

**Affiliations:** ^1^Mucosal Infection and Immunity Group, Department of Medicine, Section of Virology, St. Mary’s Campus, Imperial College London, London, United Kingdom; ^2^National Heart & Lung Institute, Imperial College London, London, United Kingdom; ^3^Respiratory Biomedical Research Unit, Royal Brompton & Harefield NHS Trust, Imperial College London, London, United Kingdom

**Keywords:** influenza, respiratory syncytial virus infections, gut microbiota, *Bacteroidetes*, *Firmicutes*, Mucin 5ac

## Abstract

Alterations in the composition of the gut microbiota have profound effects on human health. Consequently, there is great interest in identifying, characterizing, and understanding factors that initiate these changes. Despite their high prevalence, studies have only recently begun to investigate how viral lung infections have an impact on the gut microbiota. There is also considerable interest in whether the gut microbiota could be manipulated during vaccination to improve efficacy. In this highly controlled study, we aimed to establish the effect of viral lung infection on gut microbiota composition and the gut environment using mouse models of common respiratory pathogens respiratory syncytial virus (RSV) and influenza virus. This was then compared to the effect of live attenuated influenza virus (LAIV) vaccination. Both RSV and influenza virus infection resulted in significantly altered gut microbiota diversity, with an increase in *Bacteroidetes* and a concomitant decrease in *Firmicutes* phyla abundance. Although the increase in the *Bacteroidetes* phylum was consistent across several experiments, differences were observed at the family and operational taxonomic unit level. This suggests a change in gut conditions after viral lung infection that favors *Bacteroidetes* outgrowth but not individual families. No change in gut microbiota composition was observed after LAIV vaccination, suggesting that the driver of gut microbiota change is specific to live viral infection. Viral lung infections also resulted in an increase in fecal lipocalin-2, suggesting low-grade gut inflammation, and colonic Muc5ac levels. Owing to the important role that mucus plays in the gut environment, this may explain the changes in microbiota composition observed. This study demonstrates that the gut microbiota and the gut environment are altered following viral lung infections and that these changes are not observed during vaccination. Whether increased mucin levels and gut inflammation drive, or are a result of, these changes is still to be determined.

## Introduction

The bacteria that colonize the gastrointestinal tract, known collectively as the gut microbiota, play many roles in maintaining human health, such as promoting the development of the mucosa and aiding nutrient metabolism. Importantly, the microbiota protects against enteropathogen colonization through a range of mechanisms including the production of antimicrobial peptides, competition for resources, and the induction of local immune responses (1). The gut microbiota also has systemic influences outside the gastrointestinal tract, from the wide-ranging anti-inflammatory effects of bacterial metabolites like short-chain fatty acids to the alteration of neurotransmitter production in the central nervous system (2, 3). One of the most studied areas is the effect of the gut microbiota on immune responses. Germ-free mice, which lack a microbiota, have reduced expression of antimicrobial peptides, fewer antibody-secreting cells, and deficiencies in T cell function (4, 5) which result in reduced responses to influenza virus infection and vaccination (6).

Given its impact on health, research has focused on understanding what factors influence the composition of the gut microbiota. Potentially, the largest contributor to gut microbiota composition is diet. It is thought that the distinct enterotypes into which most human gut microbiotas fall are shaped by diet (7), and everything from the amount of coffee consumed to bread-type preference has been linked to gut microbiota composition (8). Similarly, medication, particularly antibiotic use, can significantly alter gut microbiota composition, and even a short course of antibiotics can have long-lasting effects (9). Infection also shapes the gut microbiota, but the majority of research has focused on gastrointestinal infections or infections that have an impact on the immune response, such as HIV (10, 11). Despite their very high prevalence, little is known about how lung infections affect the gut microbiota.

Lung infections are the leading cause of death in lower-income countries (12) and are the single biggest cause of death in children under the age of 5 (13). Respiratory syncytial virus (RSV) in particular is the most common cause of bronchiolitis and pneumonia in infants (14), and global seasonal influenza virus epidemics are thought to result in three to five million severe infections every year (15). The importance of respiratory infections on global health has led many microbiota researchers to investigate how the gut microbiota might influence lung infection; for example, the depletion of the gut microbiota in mice with antibiotics has been associated with reduced influenza virus-specific T cell and antibody generation (16). Likewise, alveolar macrophages from germ-free mice have been shown to have reduced a phagocytic capacity leading to an increased susceptibility to pneumonia (17). As the high morbidity and mortality associated with lung infections are often due to lack of effective vaccines, there is significant interest in whether the gut microbiota could be manipulated to improve vaccine efficacy as well as response to infection (18).

Whether the “gut-lung axis” is bidirectional and lung infections influence the gut microbiota is currently under debate. Several studies have been published in the last few years on the impact of influenza virus infection on the gut microbiota, but the mechanisms presented behind the changes are conflicting, and further, more in-depth, characterization studies are required. In the present study, we investigated the impact of viral lung infection on the gut microbiota using both RSV and influenza virus as infection models. We also compared this to the effect of protective live attenuated influenza virus (LAIV) vaccination on the gut microbiota as many studies looking to alter the gut microbiota before or during vaccination do not take the effect that vaccination itself may have into account.

## Materials and Methods

### Animal Experiments

Specific pathogen-free 10- to 12-week-old female BALB/c mice were purchased from Charles River Laboratories (Margate, UK). Mice were maintained in individually ventilated autoclaved cages, with Tapvei Eco Pure Premium Aspen Chips for bedding (Datesand) and Sizzle Pet for nesting material (LBS), in groups of five animals per cage. Mice were fed irradiated SDS RM3 pellets (LBS) and received reverse osmosis, autoclaved water *ad libitum*. The same specific pathogen-free room was used to house all mice and was maintained on a 12-h light/dark cycle at 20–24°C with 55 ± 10% humidity. For infection studies, mice were anesthetized *via* isoflurane inhalation and infected intranasally with 100 µl of 2 × 10^6^ PFU/ml RSV-A2, 100 µl of 4 × 10^5^ PFU/ml A/Eng/195/2009 influenza virus, or 100 µl phosphate-buffered saline (PBS). For vaccination studies, mice were anesthetized and infected intranasally with 1 × 10^6^ PFU/ml LAIV vaccine (Fluenz^®^ Tetra, MedImmune, 2016/2017 season), a dose shown to be protective in mice against challenge with 4 × 10^5^ PFU/ml A/Eng/195/2009 influenza virus. Mice were weighed daily after infection/vaccination, placed into individual disinfected pots, and feces were collected using autoclaved tweezers and stored in sterile tubes at −80°C. All animal experiments were performed in accordance with the United Kingdom’s Home Office guidelines under animal study protocol number one, and all work was approved by the Animal Welfare and Ethical Review board at Imperial College London. Studies followed the ARRIVE guidelines, and all animal infections and infectious work were carried out in biosafety level-two facilities.

### RSV L Gene qPCR

Viral load was assessed by extracting RNA from frozen lung and colon tissue disrupted in a TissueLyzer (Qiagen, Manchester, UK) using Trizol and converting it into cDNA using Omniscript RT Kit (Qiagen, Manchester, UK). RT-PCR was carried out using bulk viral RNA, for the RSV L gene and mRNA using the following primers: 5′-GAACTCAGTGTAGGTAGAATGTTTGCA-3′, 5′-TTCAGCTATCATTTTCTCTGCCAA-3′ and probe: 5′-FAM-TTTGAACCTGTCTGAACAT-TAMRA-3′ on a Stratagene Mx3005p (Agilent technologies, Santa Clara, CA, USA). RNA copy number was determined using an RSV L gene standard.

### 1 6S rRNA Gene qPCR

Bacterial DNA was extracted from 30-mg feces/mouse using the FastDNA^®^ Spin Kit for Soil (MP Biomedicals). The 16S rRNA gene was quantified using the SYBR Fast qPCR Kit Master Mix (2X, KAPA Biosystems, KK4601) and the following primers: 5′-AYTGGGYDTAAAGNG-3′, 5′-TACNVGGGTATCTAATCC-3′ (19). Reactions were performed in triplicate alongside a cloned *Vibrio natriegens* full-length 16S rRNA gene standard. Reaction plates were run on the ViiA7 Real-Time PCR System using the following run parameters: 90°C for 3 min (95°C for 20 s, 50°C for 30 s, 72°C for 30 s), 40 cycles, 10 min at 12°C.

### 1 6S rRNA Gene Sequencing

The V4 variable region of the 16S rRNA gene was amplified by PCR using 1 µl of sample DNA, Q5^®^ Hot Start High-Fidelity 2X Master Mix (NEB, M0494S), and the universal bacterial primers S-D-Bact-0564-a-S-15: 5′ AYT GGG YDT AAA GNG 3′ and S-D-Bact-0785-b-A-18: 5′ TAC NVG GGT ATC TAA TCC 3′ which were uniquely barcoded for each sample [barcodes: Illumina Nextera indexes version 2, primers: Klindworth et al. (19)]. The 16S rRNA gene library was amplified using the following run parameters: 95°C for 2 min (95°C for 20 s, 50°C for 30 s, 72°C for 5 min), 32 cycles. Each PCR was run in quadruplicate with a negative and positive control in each run. The library was purified using AMPure^®^ XP beads (Beckman Coulter, A63880) and quantified using the PicoGreen^®^ quantification assay for double-stranded DNA (Thermo Fisher, P11496). Samples were equi-molar pooled to 45 ng/sample, and the pooled library was purified again and concentrated using AMPure^®^ beads followed by agarose gel purification. Prior to sequencing, library quality was accessed by profiling using the High Sensitivity DNA Kit (Agilent Technologies, 5067-4626) on an Agilent 2100 Bioanalyzer and quantified using the illumina Library Quantification Kit. Paired-end sequencing of an 8-pM denatured library, spiked with 8 pM of PhiX, was performed using the Illumina MiSeq platform (20).

### Bioinformatics

16S rRNA-sequencing data were processed using QIIME 1.9.0 software suite (21). Sequences were trimmed, forward and reverse reads were paired, demultiplexed, and any Phix contamination removed using Burrows–Wheeler Aligner (22). Operational taxonomic units (OTUs) were clustered at 97% sequence identify using the UCLUST OTU-clustering tool (23) using open reference clustering, and representative OTUs were picked using the SILVA 115 rRNA database (24). Sequences were aligned using PyNAST (25). Chimeric sequences were identified and removed using ChimeraSlayer (26). Taxonomy was assigned using the RDP classifier (27) and the SILVA 115 rRNA database for reference sequences (24). An approximately maximum-likelihood phylogenetic tree was built using FastTree 2.1.3 (28). Microbiota analysis was conducted in R 3.3.0 (29) with RStudio (30) using the phyloseq package (31) unless otherwise specified. Beta diversity was analyzed using both the phyloseq and the vegan package (32). Beta diversity measures the difference in overall bacterial community composition between different samples. To do this, a distance matrix using the Bray–Curtis dissimilarity index, which calculates differences in bacterial OTU abundance between samples, was created and then analyzed using non-metric multidimensional-scaling (NMDS) ordination. NMDS ranks the order of inter-sample distances which is then represented by the position of samples in the two-dimensional ordination map; the closer together the two sample points are, the more similar their microbiota composition is (33). For each grouping variable, 95% confidence ellipses were calculated using the vegan package; overlapping ellipses generally indicate that microbiota composition is not significantly different although this was formally tested using Permutational Multivariate Analysis of Variance (PERMANOVA). Differences in OTU abundance were calculated using the DESeq2 package on unrarefied/untransformed data (34).

### Data Availability

Sequencing data will be uploaded to the European Nucleotide Archive under the accession number PRJEB21782. Metadata, mapping files, OTU tables, phylogenetic trees, and codes used for analysis will be uploaded to BioStudies at EMBL-EBI.

### Histology

Transverse 4-µm sections of colon and longitudinal 4-µm section of lung were cut and stained with hematoxylin and eosin (35). Airway inflammation was assessed in a blinded manner using a system similar to that used by Ponnuraj et al. (36). The degree of colonic inflammation was assessed by counting and measuring the length and width of lymphoid aggregates across the entire section at ×20 magnification using a 10-µm eyepiece graticule.

### Cytokine ELISA

Airway lavage and colon lavage fluid were collected during culling by flushing the airways and colon with 1,000 and 100 µl, respectively, of PBS. Cytokine levels in the airway and colon lavage were assessed using Mouse IFN-y, IL-13, or IL-17 DuoSets (R&D Systems, Abingdon, UK).

### Mucin ELISA

ELISAs to measure the level of Mucin 5 ac (Muc5ac) and Muc2 in the airway and colon lavage fluid were adapted from previously published protocols (37, 38). Briefly, plates were pre-incubated with carbonate buffer (pH 9.5). Airway and colonic lavage samples were then added to the carbonate buffer and incubated at 37°C overnight. Plates were blocked with 1% BSA PBS. Mucin was detected with anti-Muc5ac antibody (45M1, Thermo Fisher Scientific, UK) or anti-Muc2 antibody (ab76774, Abcam, UK). For Muc5ac, goat α-mouse HRP secondary antibody was used; for Muc2, goat α-rabbit HRP secondary antibody was used. ELISAs were developed using TMB (Thermo Fisher Scientific, UK) and stopped using 2 N H_2_SO_4_. Plates were read at 540 nm using a Fluostar (Omega).

### Fecal Lipocalin-2

Levels of fecal lipocalin-2 are considered a more sensitive marker of low-grade intestinal inflammation compared to histological analysis (39). Lipocalin-2 levels were measured in 100-mg/ml feces (reconstituted in PBS 0.1% Tween 20) using Mouse Lcn-2 DuoSet ELISA kit (R&D Systems, Abingdon, UK) as described previously (40).

### Statistics

Statistics were performed using either GraphPad Prism V6 or R 3.3.0. Two-way repeated measures analysis of variance (ANOVA) with Dunnett’s correction was used to test for significant differences in bacterial load, alpha diversity, and phyla/family abundance before infection/dosing and after. Significant changes in airway/colon inflammation and cytokine/mucin ELISAs were measured using one-way ANOVA to compare infected with PBS controls with Dunnett’s correction. Significant changes in beta diversity were calculated using PERMANOVA on the Bray–Curtis distance matrix. Using the DESeq2 package, only OTUs, which significantly changed in abundance by *p* ≤ 0.01, were selected with Benjamini–Hochberg multiple-inference correction.

## Results

### The Composition of the Gut Microbiota Is Altered following Lung Infection

**Figure 1:**
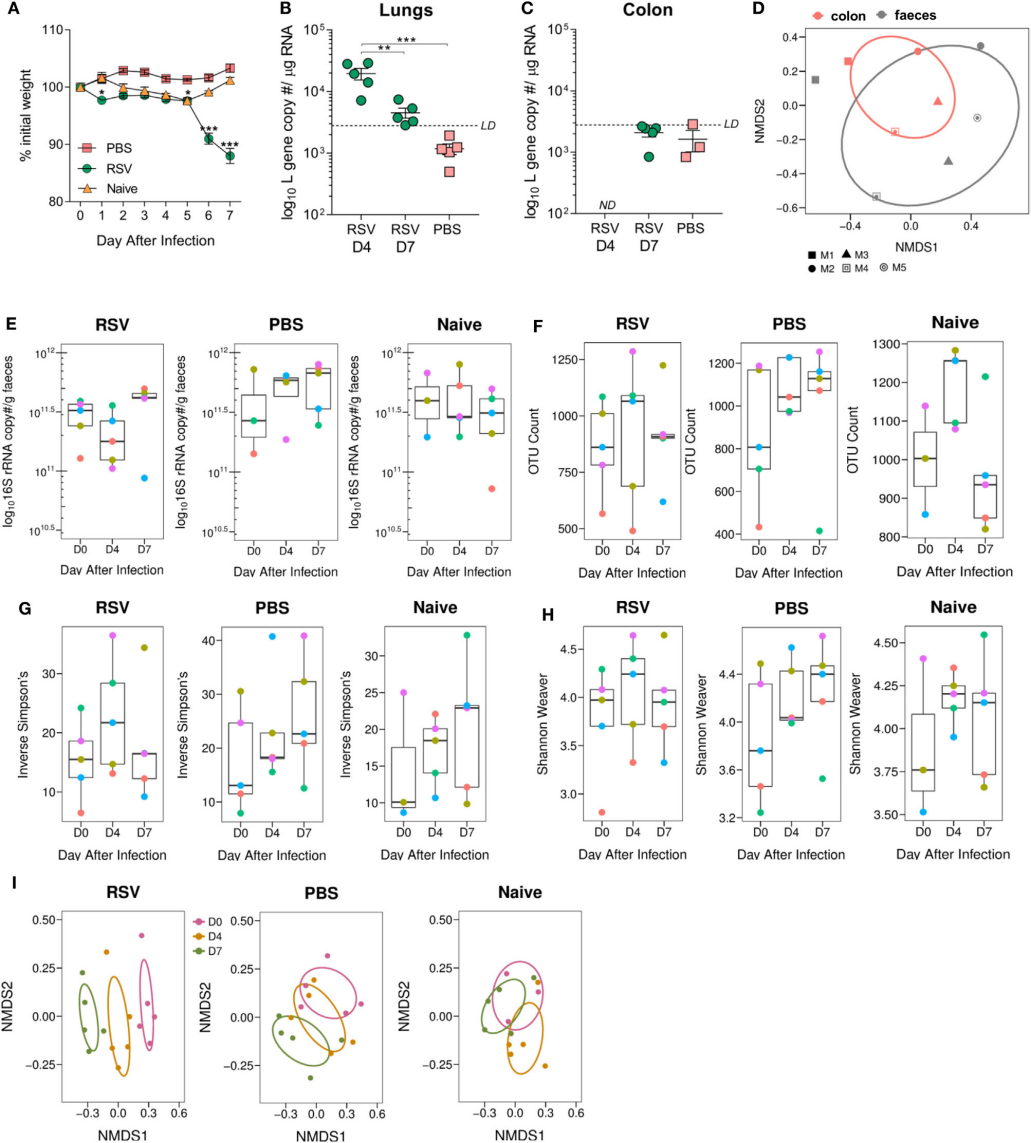
Gut microbiota diversity is altered following viral lung infection. Adult BALB/c mice were intranasally dosed with 2 × 10^6^ PFU/ml respiratory syncytial virus (RSV)-A2, sterile phosphate-buffered saline (PBS) or untreated (naïve). Feces were collected under sterile conditions before infection (D0) and at days 4 (D4) and 7 (D7) after infection. **(A)** Weight was measured after dosing. **(B)** Viral load in the lungs and colon **(C)** was estimated using RSV L gene qPCR at D4 and D7 after infection (limit of detection *LD* for the assay was 2,800 copies; not detected *ND* 0 copies/no CT value). **(D)** Colonic microbiota composition (red) was compared to the fecal microbiota composition (black) of the same mice (shapes represent individual mice). **(E)** Bacterial load in the feces was estimated using 16S rRNA qPCR. **(F)** Number of operational taxonomic units (OTUs) in feces before and after infection. **(G,H)** Alpha diversity of the gut microbiota was analyzed using the phyloseq package in R v3.4.1. **(I)** Beta diversity of the fecal microbiota was analyzed using non-metric multidimensional scaling (NMDS) on a Brays–Curtis distance matrix. *N* = 5 mice. **(E,H)** Colored points represent indicial mice. Two-way repeated measures Analysis Of Variance with Dunnett’s correction was used to test for significant differences in viral and bacterial load. Significant changes in microbiota diversity were tested for using Permutational Multivariate Analysis of Variance. *p ≥ 0.05, **p ≥ 0.01, ***p ≥ 0.001.

The aim of the study was to investigate the effect of viral lung infection on the gut microbiota. Mice were intranasally dosed with RSV, PBS, or left naïve. RSV-infected animals lost weight on days 1, 5, 6, and 7 following infection, while PBS and naïve animals experienced no significant weight loss (Figure 1A). Viral load in the airways was highest at day 4 after infection and was almost undetectable by day 7 as has been shown previously (41), despite day 7 being associated with peak weight loss for RSV-A2 infection (42) (Figure 1B). No RSV RNA was detected in the colon of infected mice at either time point (Figure 1C). To confirm that feces were a reasonable substitute for sampling the colonic environment (43), we compared the microbiota composition of colonic and fecal samples taken from the same mice (Figure 1D) and found no significant differences. Therefore, for further subsequent experiments, feces were used to monitor changes in the gut microbiota. Lung infection did not alter the total fecal bacterial load estimated using either 16S rRNA gene copy number (Figure 1E), total observed OTU (Figure 1F), or alpha diversity (Figures 1G,H). While the total bacterial load remained constant, the composition significantly changed on days 4 and 7 after RSV infection compared to day 0 (*p* = 0.006, Figure 1I). Composition changes were not observed among PBS-dosed or -naïve animals. From this, we conclude that RSV infection results in global changes to the gut microbiota.

**Figure 2:**
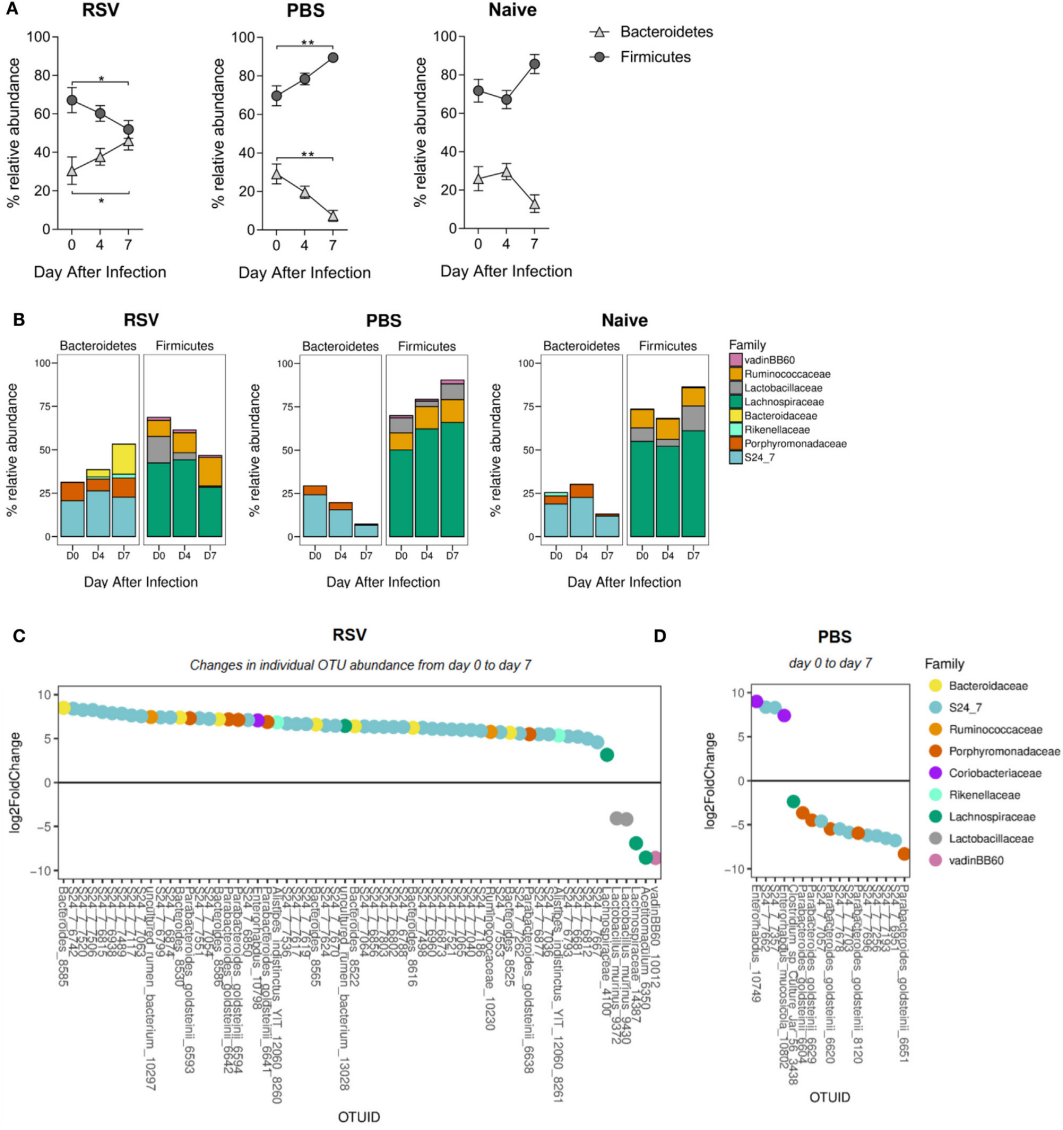
The ratio of *Bacteroidetes* to *Firmicutes* increases in the gut microbiota following respiratory syncytial virus (RSV) infection. **(A)** The relative abundance of the *Firmicutes* and *Bacteroidetes* phyla before and after RSV infection, phosphate-buffered saline (PBS) dosing and among naïve mice. Points represent the mean of *N* = 5 mice, ±SEM. **(B)** The relative abundance of each phylum splits into the most abundant gut microbiota families (only families with >1% total abundance included). **(C)** Fold change of actual operational taxonomic units (OTU) abundance after RSV infection and PBS dosing (day 7) **(D)** compared to before (*p* = 0.01 cutoff for significance). Two-way repeated measures Analysis of Variance with Dunnett’s correction was used to test for significant differences in phyla and family abundance. *p ≥ 0.05, **p ≥ 0.01.

Having seen overall diversity changes, we wished to dissect these changes at a phyla level. The dominant phyla in all mice, before and after infection, were *Bacteroidetes* and *Firmicutes* (~97–99% combined total relative abundance; data not shown). Other phyla detected were *Tenericutes, Actinobacteria, Proteobacteria*, and *Deferribacteres* but as these individually never exceeded ~2% relative abundance before or after infection, we decided to focus on changes in the dominant phyla. We observed a significant increase in the relative abundance of *Bacteroidetes* and a corresponding decrease in *Firmicutes* from days 0 to 7 after RSV infection (Figure 2A). The increase in *Bacteroidetes* after lung infection was driven by a significant increase in the relative abundance of the *Bacteroidaceae* family at day 7 (*p* ≤ 0.001; Figure 2B). Significant changes in individual bacterial OTUs belonging to the *Bacteroidaceae* and S24_7 families were also observed (Figure 2C). The decrease in *Firmicutes* was associated with a significant decrease in the relative abundance of both the *Lachnospiracea*e (*p* ≤ 0.05) and the *Lactobacillaceae* (*p* ≤ 0.01) families. While no changes were seen in naïve mice, mice dosed with PBS had an increase in the *Firmicutes* phylum and a decrease in *Bacteroidetes*, driven by an increase in the *Lachnospiraceae* family (*p* ≤ 0.01) (Figure 2D). Therefore, the composition of the gut microbiota was significantly altered following lung infection with a specific enrichment for *Bacteroidetes*.

### Changes in the Microbiota after Lung Infection Are Not Influenced by Cage Effect or Passed on to Uninfected Mice

**Figure 3:**
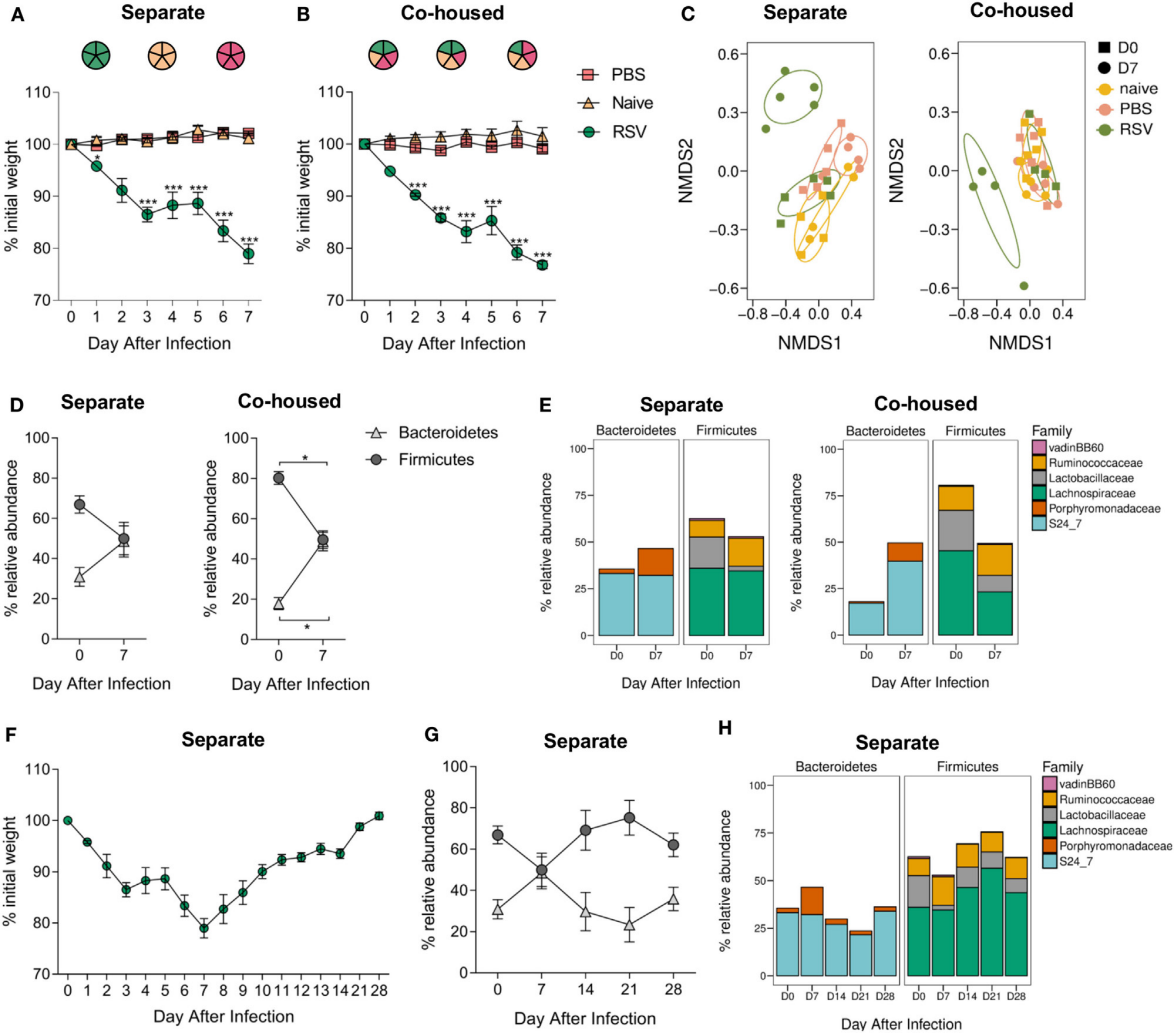
Respiratory syncytial virus (RSV) infection—associated changes in the gut microbiota are not due to cage effect or passed on to cage mates. BALB/c mice were intranasally infected with 2 × 10^6^ PFU/ml RSV-A2, dosed with phosphate-buffered saline (PBS) or untreated, animals were either housed **(A)** separately by treatment regime or **(B)** co-housed with mice receiving a different treatment. **(A,B)** Weight was measured after treatment. **(C)** Feces were collected before (D0) and after infection (D7), and beta diversity of fecal microbiota assessed. **(D)** Relative abundance in microbiota at the phyla and **(E)** family levels. **(F)** BALB/c mice infected with RSV and housed separately were allowed to recover their lost weight. **(G)** Further fecal samples were taken at D14, D21, and D28, and relative abundance at phyla **(H)** and family levels assessed. *N* = 5, points represent the mean ± SEM. Two-way repeated measures Analysis Of Variance with Dunnett’s correction for multiple comparisons was used to test for significant weight loss and changes in phyla and family abundance. *p ≥ 0.05. Non-metric multidimensional scaling (NMDS) on a Brays–Curtis distance matrix was used to visualize diversity, and significant changes in diversity were analyzed using Permutational Multivariate Analysis of Variance, comparing D0–D7 for all groups and D0 between groups.

The microbiota can be affected by a wide range of environmental stimuli and, although housing conditions were the same for all groups in this study, one potential variable was that since the infected animals were housed separately from controls, changes might be driven by cage-specific effect (44). While RSV is not passed between mice, effects on the microbiota could be transferred by coprophagy. To test whether there were cage effects and whether RSV infection-associated changes in the gut microbiota could be passed to non-infected cage mates, RSV-infected mice were mixed with PBS-dosed and -naïve cage mates. Housing infected mice separately or together with control animals had no effect on weight loss; infected animals lost weight while control animals did not (Figures 3A,B). The beta diversity of the fecal microbiota significantly changed after RSV infection for mice either separately (*p* = 0.012) or co-housed (*p* = 0.029; Figure 3C). Co-housing control mice with infected mice had no effect on the beta diversity of the control mice. Following RSV infection, there was an increase in the relative abundance of *Bacteroidetes* and a corresponding decrease in *Firmicutes* at day 7, and this was unaffected by co-housing with control animals (Figure 3D). Therefore, cage effect did not have an impact on the microbiota changes seen after RSV infection.

Interestingly, while there was an increase in the relative abundance of families belonging to the *Bacteroidetes* phylum in both the separately and co-housed mice after RSV infection, this increase was driven by the significant expansion of different families within *Bacteroidetes*. RSV-infected animals in separate cages had a significant increase in the *Porphyromonadaceae* family (*p* ≤ 0.05; Figure 3E) while infected animals co-housed in mixed groups had an increase in the S24_7 family (*p* ≤ 0.05). The reduction in *Firmicutes* phylum was associated with a significant decrease in *Lachnospiraceae* and *Lactobacillaceae* in both cases.

Respiratory syncytial virus-infected mice took 28 days after infection to return to their original starting weight (Figure 3F). Changes in microbiota were transient, preceded weight recovery, and by day 14, there was no difference in the relative percentage abundance of either phylum *Bacteroidetes* or *Firmicutes* when compared to preinfection, demonstrating the resilience of the gut microbiota (Figures 3G,H).

### Influenza Virus Infection but Not Vaccination Alters the Composition of the Gut Microbiota

**Figure 4:**
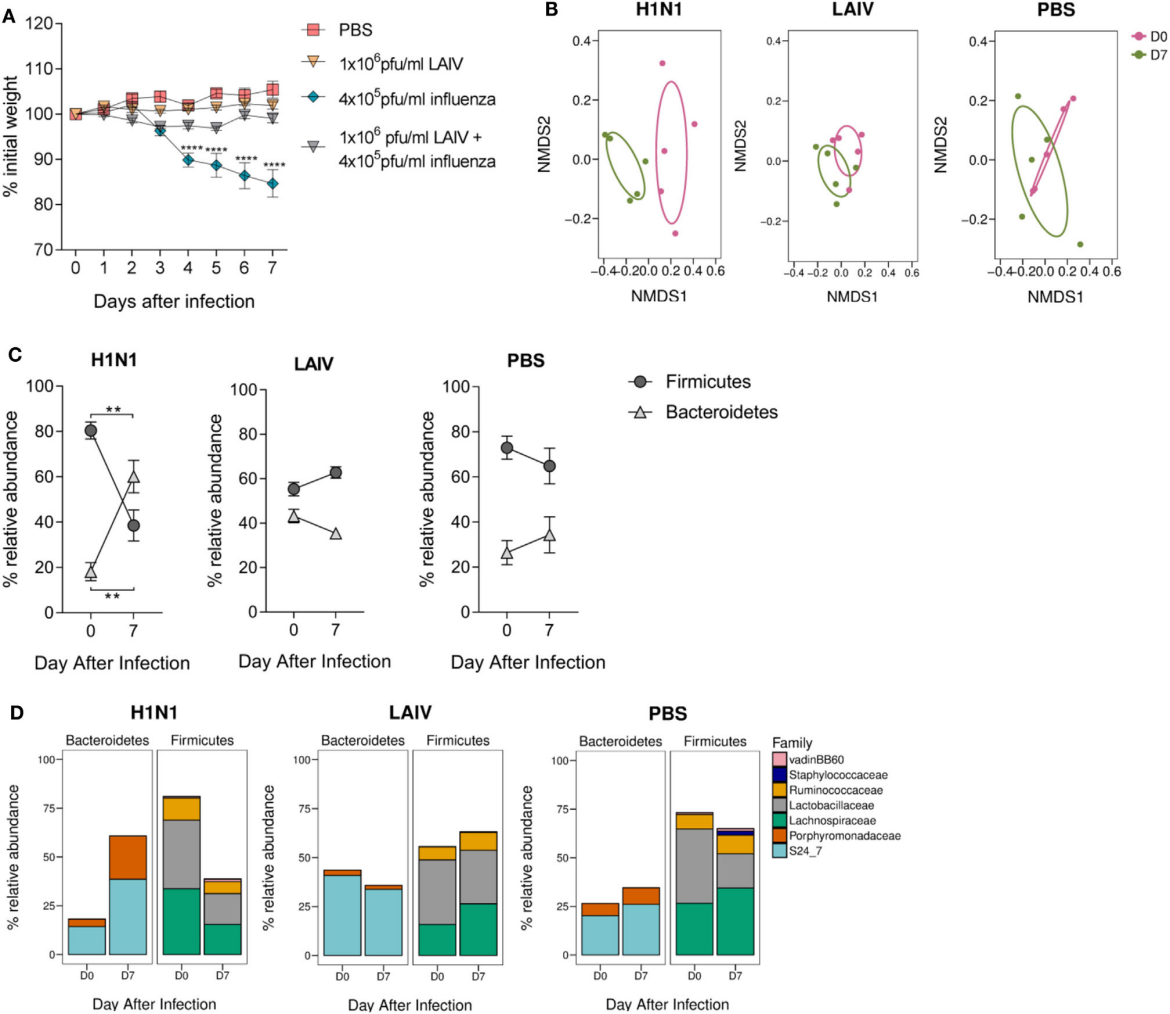
The gut microbiota is altered following influenza virus infection, but not live attenuated influenza virus (LAIV) vaccine. BALB/c mice were intranasally infected with 4 × 10^5^ PFU/ml A/Eng/195/09 influenza virus, intranasally immunized with 1 × 10^6^ PFU/ml LAIV or intranasally dosed with phosphate-buffered saline (PBS). Vaccinated mice were challenged with 4 × 10^5^ PFU/ml A/Eng/195/09 influenza virus 3 weeks later to establish that this dose of LAIV was protective against infection. Feces were collected before (D0) and after primary infection/immunization (D7). **(A)** Weight loss was recorded for 7 days after primary infection/immunization and for an additional 7 days after immunized mice were challenged. **(B)** Beta diversity of the fecal microbiota before and after influenza virus infection, LAIV immunization, and PBS dosing. **(C)** The relative abundance of the *Firmicutes* and *Bacteroidetes* phyla. **(D)** The relative abundance of *Bacteroidetes* and *Firmicutes* splits into the most abundant families (>1% total abundance) before and after influenza virus infection, LAIV immunization, and PBS dosing. *N* = 5, points represent the mean ± SEM. Two-way repeated measures analysis of variance with Dunnett’s correction for multiple comparisons was used to test for significant weight loss and changes in phyla and family abundance. *p ≥ 0.05, **p ≥ 0.01, ***p ≥ 0.001. Non-metric multidimensional-scaling (NMDS) on a Brays–Curtis distance matrix was used to visualize diversity, and significant changes in diversity were analyzed using Permutational Multivariate Analysis of Variance comparing D0–D7.

To establish whether changes observed in the gut microbiota after lung infection were RSV-specific, mice were intranasally infected with H1N1 influenza A and feces collected before and after (D7) infection. To compare the effect of infection versus vaccination on the gut microbiota, a separate group of mice were intranasally vaccinated with a dose of LAIV known to confer protection against infection in mice (Figure 4A). Mice infected with influenza had significantly altered gut microbiota diversity (*p* = 0.008; Figure 4B), whereas there was no change in gut microbiota diversity after LAIV vaccination. Influenza virus infection also led to an increase in the ratio of *Bacteroidetes* to *Firmicutes* (Figure 4C). This increase was driven by an increase in the relative abundance of the S24_7 family (*p* ≤ 0.01) and the *Porphyromonadaceae* family (*p* ≤ 0.05; Figure 4D). No changes in phyla or family abundance among vaccinated or control animals were observed (Figures 4C,D). Therefore, following both RSV and influenza virus infection, there is an increase in the abundance of the *Bacteroidetes* phylum while LAIV vaccination does not have a significant impact on the gut microbiota.

### Lung Infection Is Associated with an Increase in Low-Grade Gut Inflammation and Colonic Muc5ac Levels

**Figure 5:**
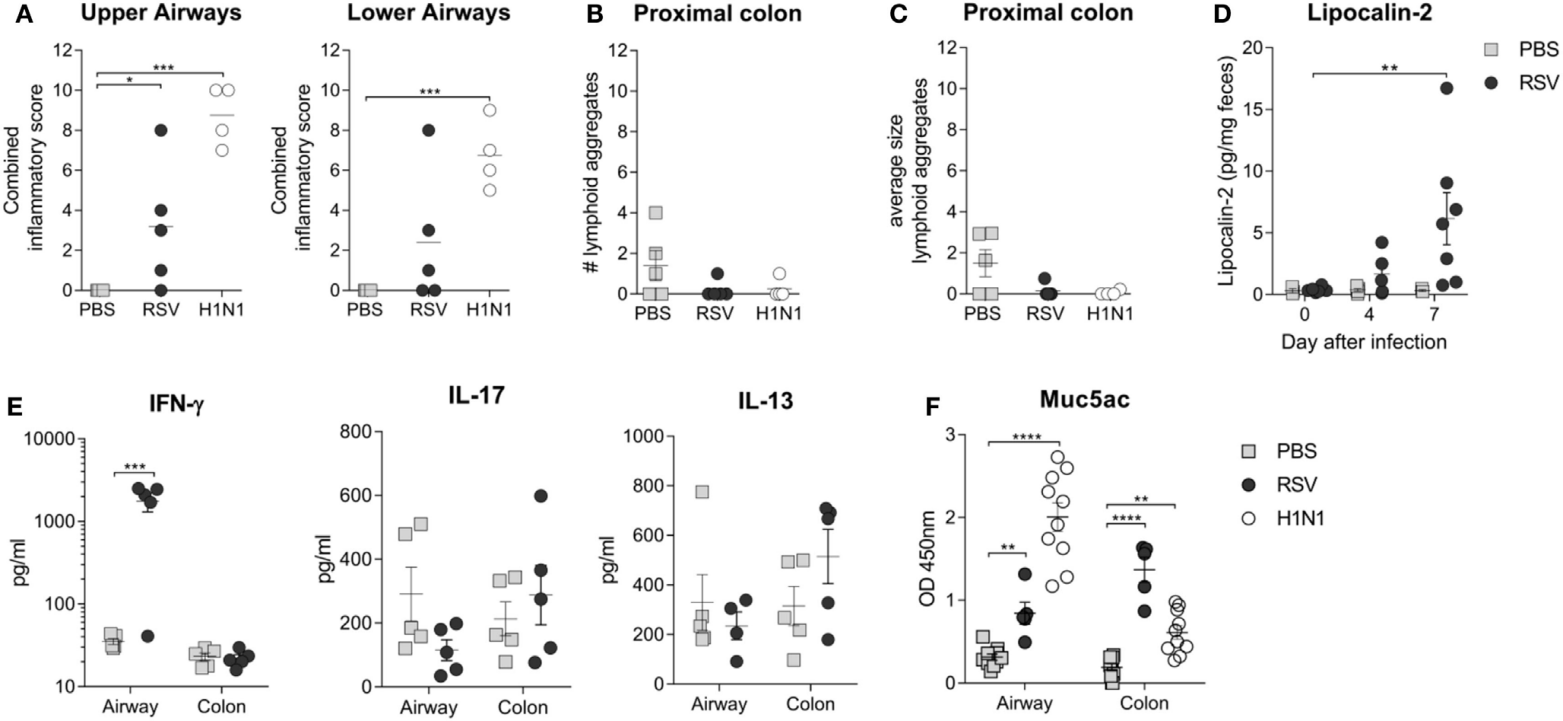
Respiratory infection results in increased colonic Mucin 5ac (Muc5ac) and low-grade gut inflammation. **(A)** Peribronchiolar, perivascular, and interstitial airway inflammation was assessed in a blinded manner for the upper and lower airways and combined together to give a combined inflammatory score. **(B,C)** Colonic inflammation was scored by counting and measuring the number **(B)** and size **(C)** of lymphoid aggregates in the colonic epithelium. **(D)** Low-grade gut inflammation was assessed by measuring fecal lipocalin-2 levels. **(E)** IL-17, IL-13, and IFN-y cytokine levels were measured in the bronchoalveolar lavage fluid (airways) and colonic lavage fluid (colon) after respiratory syncytial virus (RSV) infection or phosphate-buffered saline (PBS) dosing. **(F)** Muc5ac levels were measured in the airway and colon after RSV infection, H1N1 infection, and PBS dosing. *N* = 5–10 mice/group ± SEM, two-way analysis of variance. *p ≥ 0.05, **p ≥ 0.01, ***p ≥ 0.001.

Previous studies investigating the connection between the gut microbiota and lung infections have focused on the exploration of potential immunological mechanisms (16, 17, 45). In the present study, significant upper airway inflammation after RSV infection and significant upper and lower airway inflammation after influenza virus infection were observed (Figure 5A). However, there was no evidence of any significant histological colonic inflammation after either RSV or influenza infection (Figures 5B,C). Fecal levels of lipocalin-2 are considered a more sensitive marker of gut inflammation that histological analysis (39); we observed significantly higher fecal lipocalin-2 levels after RSV infection, suggesting that viral lung infections may result in low-grade gut inflammation (Figure 5D). To explore this further, levels of cytokines in airway and colonic lavage were measured after RSV infection. IFN-γ was elevated after RSV infection in the airways but not in the colonic lavage, and no significant increases in levels of IL-13 or IL-17 at either site were found (Figure 5E). RSV and influenza viruses, similar to most respiratory pathogens, cause elevated mucus secretion in the airways. As mucus is nutrient rich, raising its level in the guts either by swallowing airway mucus or by systemic hypersecretion could explain the bloom in certain microbiota members. Many members of the gut microbiota, including members of the *Bacteroidetes* phylum, use mucus as an energy source (46). Interestingly, Muc5ac levels were significantly increased in both the airways and the colon of RSV or influenza virus-infected mice but not in those of control mice (Figure 5F). These findings suggest that the changes in gut microbiota composition observed after both lung infections could be due to the mucus hypersecretion induced by both respiratory viruses.

## Discussion

In the current study, we demonstrate that the composition of the gut microbiota changes after viral lung infection. This study also contributes to understanding the complex relationship between the gut microbiota and influenza infection and, for the first time, characterizes how the gut microbiota is altered following RSV infection. Viral lung infection led to an increase in the phylum *Bacteroidetes* with a corresponding decrease in the *Firmicutes* phylum. The constituent bacteria that drove these changes varied at the family and OTU levels, suggesting that viral lung infection creates conditions favorable to support a *Bacteroidetes* bloom, but that the species that make up this bloom are selected by currently unknown factors. In addition, we observed no change in gut microbiota diversity or composition after LAIV vaccination, emphasizing that the observed changes are dependent upon infection and providing a preliminary baseline for future studies investigating the impact of vaccination on the gut microbiota and *vice versa*.

As in humans, the majority of the murine gut and fecal microbiota belongs to either the *Firmicutes* or *Bacteroidetes* phyla (47, 48), and a change in the balance between the two has been previously implicated in many diseases and disorders (49). We observed an increase in *Bacteroidetes* and a decrease in *Firmicutes* in the gut microbiota of mice following lung infection. A decrease in the relative abundance of *Firmicutes* has been reported in a chronic mild stress model in mice, with a specific reduction of gut *Lactobacillus* (50), suggesting that changes in gut microbiota composition observed by us and others may be more reflective of a stress response. A decrease in *Lactobacillus*, detected by PCR, has previously been observed after influenza infection (51), and in this study, we consistently saw a decrease in the *Lactobacillaceae* family after viral lung infection. Other studies investigating the effect of influenza virus in mice have highlighted an increase in the *Proteobacteria* phylum after infection (52, 53). In our study, the relative abundance of *Proteobacteria* never exceeded ~0.01%, and there was no change after infection. Although no significant increase in the relative abundance of the *Bacteroidetes* phylum was observed in these previous studies, the abundance of OTUs belonging to *Bacteroidetes* correlated most significantly with weight loss after influenza infection (52). No comparative study in humans investigating the direct effect of lung infections on the gut microbiota has currently been published. However, one group, which profiled the gut microbiota of infants with and without bronchiolitis, found that there was a strong positive association between a high *Bacteroidetes* abundance in the gut microbiota and bronchiolitis, and, interestingly, 65% of these bronchiolitis cases were RSV-positive (54).

The changes that have been observed in the gut microbiota occurring after lung infection remain unclear. Studies in mice have suggested that the immune response to influenza virus infection in the lungs shapes gut microbiota composition, with both type I (53) and type II interferons (51) proposed to have a role. Our observation that different families within the *Bacteroidetes* phylum increase in abundance suggests that rather than the specific targeting of certain microbiota members by the immune system, respiratory infection causes a change in the gut environment, favoring the expansion of *Bacteroidetes*, and whichever *Bacteroidetes* family gains the advantage first increases in abundance. We speculate that one factor contributing to these changes is mucus. We observed increased levels of mucin Muc5ac in the colon, where it is not normally expressed (55, 56). This increase may be due to mice swallowing the excess mucus produced in the airways after infection or it may be that Muc5ac expression in colonic goblet cells is stimulated by the viral infection. The main role of mucus is defense against microbial exposure, but mucus is also utilized by these same microbes to gain an advantage in the extremely competitive microbiota environment (57). Bacteria, which can use mucins as an energy source, may have an ecological advantage if mucus composition changes. This has been seen elsewhere; changes in vaginal microbiota composition were associated with increased cervical Muc5B and Muc5ac levels (58) but the impact of respiratory mucus on the gut microbiota has not been previously studied.

An alternative explanation for the changes in microbiota composition observed after lung infection is infection-induced weight loss. Diet is the biggest contributor to microbiota composition (59), and reduced calorific intake in humans has been associated with a significant increase in *Bacteroidetes* abundance over *Firmicutes* (60, 61), similar to what was observed in this study after both RSV and influenza virus infection. In addition, calorie reduction in conjunction with influenza infection has been shown to enhance the gut microbiota changes observed after influenza infection alone (52). *Bacteroidetes* are considered very metabolically flexible (62), and mouse models of nutrient deprivation have been shown to switch their gene expression profile from enzymes capable of metabolizing dietary polysaccharides to enzymes which break down host mucus glycans (63). Therefore, it may be that the increase in *Bacteroidetes* observed after lung viral infection is due to a reduced food intake, and the increase in mucus observed in the gut may be compensatory for increased mucus metabolism by the gut bacteria.

One interesting feature of this study was the increase in S24_7 abundance observed after lung infection. Despite being a very common constituent of the murine gut microbiota, only one member of this strictly anaerobic family has been isolated and cultured: *Muribaculum intestinale* (64). There is some controversy about whether S24_7 is mouse-specific (65) or part of the gut microbiota in humans (66). Equally, the impact of S24_7 on health is unclear; while some studies have associated increased S24_7 levels with inflammation, there are conflicting theories on whether S24_7 is the cause of (67) or the response to (40) the inflammation. Supporting our hypothesis that changes in gut microbiota after lung infection are driven by elevated mucus, the increased abundance of both S24_7 and the mucin-degrading bacteria *Akkermansia muciniphilia* has been observed in a gut infection/inflammation model (40), suggesting that S24_7 has a mucin-degrading ability. Likewise, the genetic analysis of S24_7 has revealed that some members of S24_7 have a tropism for host glycans (such as mucin) (66). In future studies, it will be interesting to determine the role of this family.

While the changes observed in the present study are robust and reproducible, the functional implications of the shift in microbiota after viral lung infection remain unclear. Changes in the gut microbiota have been associated with, and may in some circumstance amplify, disease. The decrease in *Lactobacillus* seen in the chronic stress model in mice (50) was associated with increased kynurenine, which is associated with depression. Changes in microbiota may also drive the gastrointestinal symptoms associated with influenza infection (68). We did observe an increase in lipocalin-2 in the feces which is associated with gut inflammation. These could be attributed to viral infection of the gastrointestinal tract, but in our study, and in others, no viral RNA was detected in the colonic tissue (51). If changes in microbiota were found in future studies to be associated with increased disease, therapeutic restoration of the preinfection balance may reduce disease. The majority of studies in humans and mice looking to improve the immune response and ameliorate disease in influenza virus infection have used various *Lactobacillus* spp. as probiotics with some success (18). Enriching the gut microbiota for *Lactobacillus* has also been shown to protect against airway inflammation in RSV infection (69). Overall, demonstrating that viral lung infection changes the gut microbiome is an important first step to investigating how these changes might have an impact on both respiratory and gut health, both in an infection setting and during vaccination.

## Ethics Statement

This study was carried out in accordance with the recommendations of the UK Home Office guidelines. The protocol was approved by the Imperial College London Animal Welfare and Ethics committee and followed the Animal Research: Reporting of *In Vivo* Experiments.

## Author Contributions

HG performed experiments, analyzed data, and wrote the manuscript. LC and PJ analyzed data. MM designed studies and wrote the manuscript. MC designed studies, analyzed data, and wrote the manuscript. JT designed studies and wrote the manuscript.

## Conflict of Interest Statement

The authors declare that the research was conducted in the absence of any commercial or financial relationships that could be construed as a potential conflict of interest.
